# Periovulatory neurohormone dynamics reveal an association between secretoneurin and GnRH across the mouse estrous cycle

**DOI:** 10.3389/fendo.2025.1708570

**Published:** 2026-01-23

**Authors:** Chunyu Lu, Di Peng, Kevin B. Smith, Chinelo Uju, Suraj Unniappan, Paula Duarte-Guterman, Nafissa Ismail, Vance L. Trudeau

**Affiliations:** 1 Department of Biology, University of Ottawa, Ottawa, ON, Canada; 2 School of Psychology, University of Ottawa, Ottawa, ON, Canada; 3 Department of Veterinary Biomedical Sciences, Western College of Veterinary Medicine, University of Saskatchewan, Saskatoon, SK, Canada; 4 Department of Psychology, Brock University, St. Catharines, ON, Canada

**Keywords:** arginine vasopressin, estradiol, gonadotropin-releasing hormone, luteinizing hormone, oxytocin, progesterone, secretogranin 2

## Abstract

**Introduction:**

Surge release of luteinizing hormone (LH) from the pituitary is essential for fertility, as it triggers ovulation. Secretoneurin (SN), a conserved peptide derived from secretogranin-2, stimulates LH release, but its relationship to periovulatory changes in classical reproductive hormones remains unclear.

**Methods:**

We measured fluctuations of two reproductive steroids and three peptides in the hypothalamus, pituitary, and ovary of female mice during the periovulatory period using a novel nano LC-MS/MS protocol. Immunohistochemistry for SN and GnRH was performed on brain samples. GT1-7 GnRH neuronal cells were exposed to various doses of SN and processed for PCR determination of Gnrh1 mRNA levels. A subset of these samples was subjected to RNA sequencing for pathway analysis.

**Results:**

P4, E2, AVP, GnRH1, and SN varied across the cycle, whereas OXT was relatively stable. Ovarian P4 was highest at proestrus, while E2 peaked at diestrus. Hypothalamic P4 was elevated at diestrus, whereas pituitary P4 remained low and variable. AVP peaked at proestrus in both the hypothalamus and pituitary. SN levels were highest in the hypothalamus and pituitary at proestrus. GnRH1 increased in the hypothalamus and pituitary at proestrus but was undetectable in ovary. Regression analysis revealed a moderate positive association between hypothalamic SN and GnRH1. SN-immunoreactive fibers were found near GnRH neurons in the median and medial preoptic areas. *In vitro*, SN increased *Gnrh1* mRNA in GT1-7 cells in a time- and dose-dependent manner. RNA sequencing after 6 h SN treatment highlights key signaling cascades including MAPK, transcriptional regulation, and calcium signaling pathways. Six upregulated TFs predicted to bind to the mouse *Gnrh1* promoter were also linked to significant enrichment in ribosome-related processes, protein synthesis, and cellular component organization pathways.

**Conclusion:**

These findings identify a periovulatory association between SN and *GnRH1* and provide a foundation for targeted studies needed to test causality in the mammalian reproductive neuroendocrine network.

## 1 Introduction

The neuroendocrine control of ovulation is fundamental for successful reproduction. Rodent and fish models have been essential for understanding how neuropeptides regulate the pulsatile and surge release of gonadotropin-releasing hormone (GnRH), which in turn drives pituitary luteinizing hormone (LH) secretion (1–4). During the periovulatory period, a surge of LH later induces ovulation. Over the past two decades, attention has focused mainly on the mammalian kisspeptin system, which is now considered a critical factor that regulates GnRH neurons and mediates the feedback actions of ovarian steroids (5–7). Often ignored, the nonapeptides oxytocin (OXT) and arginine vasopressin (AVP) act beyond their roles in parturition, lactation, and osmoregulation (8–10). Blocking OXT prevents the proestrus LH surge (11), and OXT stimulates GnRH release from hypothalamic explants (12, 13), consistent with OXT receptor expression in some GnRH neurons (14). Similarly, AVP infusion triggers an LH surge in rats (15), with peak sensitivity at proestrus when estradiol (E2) is high, and AVP receptors in GnRH neurons of female mice (16) support a direct action.

In addition to these classical regulators, evidence suggests that secretoneurin (SN) also participates in reproductive control. SN is a 31–34 amino acid peptide derived from the selective processing of the secretogranin-2 (SCG2) precursor and is well conserved from fish to mammals (17). SN is of particular interest because it colocalizes with OXT and AVP in the hypothalamo-neurohypophysial system (18, 19), and because the hypothalamus displays SN-immunoreactive fibers and neuronal perikarya (20), including in the preoptic area where critical GnRH neurons are located (1, 2). Functional studies strongly support a reproductive role for SN: in goldfish, secretoneurin A (SNa) stimulates LH production and release (21), and mouse SN similarly promotes LH secretion in LβT2 gonadotroph cells (22). TALEN-mediated frameshift mutations of zebrafish *scg2a* and *scg2b* genes severely impair reproduction, a defect partially rescued by *in vivo* injections of SNa (23). These findings suggest an evolutionarily conserved role of SN in reproductive regulation.

Despite this evidence, the physiology of the secretograninergic system remains poorly understood, particularly in mammals. Therefore, our objective was to determine if SN levels vary in relation to classical neuropeptides and steroids during the mammalian ovulatory cycle. We focused on tissue content to enable simultaneous measurement of multiple peptides and steroids within the same individuals in the whole hypothalamus, pituitary, and ovary of mice. Most prominent was that hypothalamic SN and GnRH1 peptides increased together at proestrus. This observation led us to determine that SN stimulated *Gnrh1* mRNA in mouse GT1–7 cells, and enhanced pathways associated with signal transduction, secretion, and transcriptional regulation. Our findings associate SN with periovulatory neuroendocrine dynamics in mice and motivate future *in vivo* tests of SN actions within the GnRH neuronal network.

## 2 Materials and methods

### 2.1 Chemicals

Peptides (**Table 1**) were synthesized in-house using an Intavis Multipeptide RSi peptide synthesis machine (Köln, Germany) with standard Fmoc solid-phase chemistry and purified (>95%) as described (24). Products were purified on a 4.6 × 150 mm Phenomenex Luna Omega C18 column (5 μm; Phenomenex, Torrance, CA, USA) using an Agilent 1100 HPLC system. Peptide identity and purity were verified by nano-flow UHPLC–MS/MS with a 75 μm × 100 mm analytical column packed in-house with reverse-phase Magic C18AQ resin (1.9 μm; 120 Å pore size; Dr. Maisch GmbH, Ammerbuch, Germany). This analysis confirmed that all samples were free of significant chiral isomer contamination. Solvents (LC-MS grade) were obtained from Fisher Scientific (Waltham, MA, USA). 17β-estradiol (E2) and progesterone (P4) were purchased from commercial suppliers (**Table 1**).

**Table 1:** Standards used in Nano LC-MS/MS.

Compound	Sequence or supplier information
OXT	H-CYIQNCPLG-CONH2 [Disulfide C1-C6]
AVP	H-CYFQNCPRG-CONH2 [Disulfide C1-C6]
GnRH1	Pyr-HWSYGLRPG-CONH2
SN	H-TNEIVEEQTYPQSLATLESVFQELGKLTGPSNQ-CONH2
E2	Sigma-Aldrich; Cat. # E1024
P4	Cayman Chemicals; Cat. # 15876

### 2.2 Tissue sample collection for hormonal measurements

**Figure 1:**
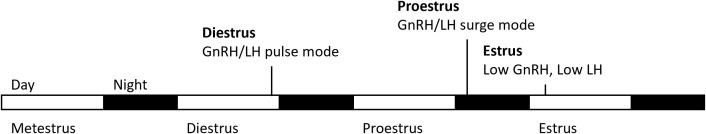
Illustration of the mouse estrous cycle relative to the light/dark cycle. The diestrus, proestrus and estrus sampling times are indicated by the vertical lines.

Female CD1 mice were obtained from Charles River Laboratories (St-Constant, Québec, Canada) at 3–5 weeks of age and maintained according to standard practices approved by the University of Ottawa Animal Care and Veterinary Services. Mice were housed in all-female rooms in groups of 4 per cage in polycarbonate cages measuring 17 cm × 28 cm × 12 cm (W × L × H) containing corn cob bedding, one cardboard hut, and one piece of nestlet. Mice had ad libitum access to food (Teklad Global Diets, Envigo) and water. The vivarium was kept on a 14 h light/10 h dark cycle (lights off at 10:00 a.m., lights on at 8:00 p.m.) at 24 ± 2 °C and 40% ± 5% humidity. Dusk and dawn transitions were gradually induced over 1 h. All procedures were approved by the Animal Care Committee of the University of Ottawa. Estrous cycle stage was determined in 10-week-old females by vaginal lavage with sterile phosphate-buffered saline (PBS) followed by cytological staging (25). Lavages were collected every morning at 09:00 to monitor cycle progression, and females were required to display at least one complete estrous cycle prior to euthanasia to ensure normalized and predictable staging. Mice (n = 9 per time point) were sampled at CO_2_ asphyxiation at three stages: (i) 1 h before lights off in diestrus, (ii) 1.5 h after lights off in proestrus, and (iii) 2 h after lights on in estrus (**Figure 1**). Euthanasia was performed using a rapid fill CO_2_ protocol consistent with institutional guidelines. Spinal transection and dissection began immediately after confirmation of euthanasia. All time points used the same euthanasia and dissection workflow to minimize bias between cycle stages. The hypothalamus, pituitary, and ovaries were rapidly dissected in <2 min. The whole brain was rapidly removed, placed ventral side up and sectioned by making coronal cuts with a single edge razor blade just anterior to the optic chiasm and posterior to the mammillary bodies. The hypothalamic block was then isolated by dissecting laterally along the hypothalamic sulci, encompassing the region between the third ventricle and optic tracts. All tissue were placed in homogenization buffer and processed immediately for analysis by validated LC-MS/MS (24). These time points were selected based on Herbison’s models of GnRH pulse and surge generator activities and corresponding LH secretion patterns and ovarian activity in female mice (6, 26).

### 2.3 Solid-phase extraction and Nano LC-MS/MS

Solid-phase extraction (SPE) was performed using in-house prepared cartridges with 10 mg sorbent (24). Before loading, the SPE bed was conditioned with 5 × 200 µL of 50% acetonitrile and equilibrated with 5 × 200 µL of 5% acetonitrile. Samples were loaded in 5% acetonitrile, washed, and eluted with 75% acetonitrile.

We followed previously published LC-MS/MS methods (24) with minor modifications, and analytes measured are listed in **Table 1**. The nano LC-MS/MS system consisted of an Agilent 1100 nanoflow HPLC (G1376A binary capillary pump, G1477A micro well-plate autosampler, G1330A ALS thermostat) coupled to a Thermo Scientific Orbitrap Velos Pro™ mass spectrometer. The LC was run in micro mode with a pre-column splitter in load and elute modes. The analytical column (50 µm × 150 mm) was packed in-house using 3 µm C18-AQ sorbent (Dr. Maisch GmbH, Ammerbuch, Germany). For positive-mode analysis, the mobile phases were: (A1) 0.2% formic acid in water, and (B1) 0.2% formic acid in 80% acetonitrile/20% water. For negative-mode analysis, the mobile phases were: (A2) 1 µM NH_4_F in water, and (B2) 1 µM NH_4_F in 65% methanol/30% acetonitrile/5% water. The positive-mode gradient was 20–90% B1 over 30 min; the negative-mode gradient was 50–90% B2 over 15 min. The loading flow rate was set at 6000 nL/min and the eluting flow rate at 300 nL/min for both positive and negative mode analyses.

The mass spectrometer operated in data-dependent acquisition mode. The Orbitrap analyzer acquired master scans at 60,000 resolutions (FWHM), and the linear ion-trap analyzer performed fragmentation and secondary MS scans according to the mass list and master scan. Data acquisition and analysis were performed with Thermo Xcalibur software (version 2.2). Quantification was determined using calibration curves and performed using Thermo Xcalibur 2.2 SP1 Quan Browser, with automatic peak regression based on Gaussian fitting, applying a signal-to-noise ratio (S/N) threshold of 15 (e.g., < 15 was considered not detected) and a retention time window of ±30 second as reported (24). The lower limit of quantification (fmol/tissue) for the analyzed compounds were: E2 (244), OXT (488), P4 (171), AVP (977), GnRH1 (15), and SN (75,000). For any measured levels below the lowest point of calibration, the standard curves were extended for semi-quantitative estimation.

### 2.4 Cell culture

Murine hypothalamic GT1–7 cell culture was carried out as previously described (27). Briefly, GT1–7 cells at passage 20 were plated onto three 24-well plates in 500 µL complete growth medium. Once cultures reached ~75% confluency, the medium was replaced with either fresh medium alone (control) or medium containing 0.1, 1, 10, 100, or 1000 nM mouse SN (AnaSpec Inc., Fremont, USA; Cat.# AS-62673; Lot# 2057426). Cells were incubated for 1, 3, or 6 h (n = 4 wells per treatment), with one plate assigned to each time point. The dose range of SN was based on earlier work reporting on effective concentrations stimulating LH release from mouse LβT2 cells (22). At the end of incubation, medium was removed, and 500 µL RiboZol™ (Cat# 1B1304-200ML, VWR Life Science, USA) was added to each well. Total RNA was extracted as described below. All experiments were conducted between 09:00–16:00 h. The experiment was independently repeated once using cells at the same passage, and results from both experiments were pooled (n = 8 per treatment).

### 2.5 Quantification of mRNA abundance by RT-qPCR

Total RNA from incubated GT1–7 cells was extracted using RiboZol™ reagent (Cat# 1B1304-200ML, VWR Life Science, USA). RNA purity was assessed by the optical density (OD) 260/280 ratio using a NanoDrop 2000c spectrophotometer (Thermo, Vantaa, Finland). cDNA was synthesized from 1 µg of total RNA in a 20 µL reaction volume using iScript Reverse Transcription Supermix for RT-qPCR (Cat# 1708841, Bio-Rad, Mississauga, ON, Canada) according to the manufacturer’s instructions. Target (*Gnrh1*) and reference (*Actb*) genes were amplified using previously validated primers (27). RT-qPCR reactions were prepared in 96-well plates with iQ™ SYBR^®^ Green Supermix (Cat# 1708884, Bio-Rad, Mississauga, ON, Canada), 1 µL cDNA, and 500 nM of each primer, in a final volume of 10 µL. Each run included a standard curve for the corresponding gene, along with no-reverse transcriptase, no-template, and water-only controls to confirm reagent integrity and exclude contamination. Cycling conditions were 95°C for 3 min, followed by 35 cycles of 95°C for 30 s and 60°C for 30 s. A melting curve analysis was performed at the end of each run to verify amplification specificity. Reactions were run on a CFX Connect Real-Time PCR System (Bio-Rad, Mississauga, ON, Canada). Relative mRNA expression was calculated using the 2−ΔΔCt method.

### 2.6 Statistical analyses

Tissue levels of peptides and steroids, and *Gnrh1* mRNA abundance data were analyzed using one-way ANOVA followed by Tukey’s *post-hoc* test. Statistical significance was considered at P < 0.05. GraphPad PRISM version 9.5 (GraphPad Inc., USA) was used for statistical analysis and figure generation. Peptide and steroid levels were not corrected for tissue weight and are reported as fmol/tissue. Simultaneous extraction and quantification of peptides and steroids in single tissue samples from mature females across the time-course permitted calculation of regression coefficients (R²). Linear regression analysis was performed to determine potential relationships between SN and reproductive hormones across the three stages within each individual female.

### 2.7 DNase treatment and library preparation for RNA sequencing

We focused on 6 h samples for control (n=6) and 100 nM SN (n = 6) for RNASeq because the PCR data revealed that the highest *gnrh1* mRNA levels in response to SN were at this time. Residual DNA was removed using DNase I (NEB, Cat# M0303S) followed by Monarch RNA Cleanup Kit (NEB, Cat# T2030L). RNA quality was assessed using the Qubit RNA HS Assay (Thermo Fisher, Cat# Q32852) and RNA ScreenTape (Agilent, Cat# 5067-5576). Libraries were generated from 50–400 ng RNA (RIN ≥ 8) using NEBNext Poly(A) mRNA Magnetic Isolation and Ultra II Directional RNA Library Prep Kits. Libraries were evaluated with Qubit dsDNA HS and D1000 ScreenTape. Pooled libraries were sequenced (75 bp paired-end) on a NextSeq 550 (Illumina) at the University of Saskatchewan Next-Generation Sequencing Facility. Reads were processed with BaseSpace (Illumina), trimmed with fastp (28), and aligned to *Mus musculus* genome assembly MM10 with STAR (29).

### 2.8 Differential gene expression and gene ontology analyses

RNA sequencing counts were batch-corrected using ComBat-seq (30) and processed with the Rsubread package (31). Differential expression was assessed using DESeq2 (32) with filtering criteria of p<0.05 and |log2FC| > 0.4. Given our qPCR showing that SN upregulates *Gnrh1* mRNA in GT1–7 cells, we extended this analysis to bulk RNA sequencing to identify potential SN-responsive genes and their potential transcriptional regulators. Pathway enrichment analysis was conducted with g:Profiler (p<0.05). Two thematic analyses were performed: (1) intracellular signaling pathways were identified using keywords such as MAPK, PI3K, Akt, cAMP, mTOR, Calcium, Wnt, and Notch. (2) secretion-related processes were identified using keywords including biosynthetic, hormone, peptide, secretion, exocytosis, vesicle, and transport (See **Supplementary File 1**). Pathways were plotted as fold change against –log10(p-values). Fold-change was calculated as the proportion of input DEGs that belong to each pathway term (intersection_size/query_size). Bioinformatic visualizations included volcano and pathway enrichment bar plots generated with `ggplot2` in R. Methods (with relevant references) used to investigate candidate regulatory relationships are presented in **Supplementary File 2**.

Upregulated transcription factors (TFs) were identified from DEGs using AnimalTFDB database annotation, with filtering criteria of p<0.05 and |log2FC| >0.4 (See **Supplementary File 1**). The *Gnrh1* gene promoter sequence (-2000 to +200 bp) was extracted and analyzed using TFBSTools package combined with JASPAR 2020 database for TF binding site prediction. The threshold was set at 85% maximum score, resulting in 6 validated TFs (ASCL2, MYC, MAFF, HES1, NRLH4, VSX1). The MEME Suite FIMO tool (p<1e-4) was used to scan promoter regions of 495 upregulated genes, identifying binding sites for the 6 TFs. Transcription factor-target gene regulatory relationships were established based on binding strength and statistical significance. The 387 genes with multiple TF binding sites were selected for pathway enrichment analysis using clusterProfiler package for GO and KEGG pathway analysis (p.adj < 0.05).

### 2.9 Mouse GnRH1 and SN immunohistochemistry

Adult female C57BL/6J mice (10 weeks; Jackson Laboratory) were maintained according to standard practices approved by the Brock University Animal Care Committee. The vivarium was kept on a 12 h light/12 h dark cycle (lights on 08:00, lights off 20:00) at 23 ± 1°C and 40 ± 5% humidity, with dusk and dawn transitions over 1 h. Mice were housed in ventilated polysulfone cages (GM500; Techniplast) in groups of 4 per cage measuring 20 cm × 39 cm × 16 cm (W × L × H) and containing corn cob bedding (Envigo), Crink-l’ Nest (The Andersons Inc.), Nestlets (Ancare), and a mouse igloo (BioServ made of high temp polycarbonate). Mice were fed a standard mouse chow (2014 Teklad Global, 14% protein rodent maintenance diet, Harlan Tekland). Estrous cycle stage was determined by vaginal lavage with sterile phosphate-buffered saline (PBS) and cytological staging (25). Samples were collected between 09:00 and 12:00 daily to ensure consistent staging. Four mice identified in proestrus were used.

Mice were anesthetized with isoflurane and perfused transcardially with ice-cold 0.9% saline, followed by 4% paraformaldehyde (PFA) in PBS. Brains were extracted, post-fixed for 24 h at 4°C, then cryoprotected in 30% sucrose at 4°C. Coronal sections (40 µm) were collected from Bregma +2.0 to −2.92 (33) and stored at −20°C in antifreeze solution (0.1 M PBS, 30% ethylene glycol, 20% glycerol). Free-floating sections were rinsed three times for 10 min in tris-buffered saline (TBS; 0.1 M, pH 7.4) between each step unless noted. Sections were blocked in 5% normal goat serum (NGS), 0.5% Triton X-100, and 1% bovine serum albumin (BSA) in TBS for 90 min. They were then incubated for 48 h at 4°C with validated guinea pig anti-GnRH (GA04; 1:5000 (34)) and validated rabbit anti-SN (1:625 (20). The antibody specifically detects mammalian SN and several larger proteins derived proteolytically from SCG2 containing the SN antigen sequence (mouse SCG2 184-216; NP_033155.1) as shown by immunohistochemistry with preabsorption assays, immunoblotting and radioimmunoassay in numerous tissues and cell lines in rats and mice (20, 22, 35, 36). After rinsing, sections were incubated with secondary antibodies: goat anti-guinea pig IgG Alexa Fluor 488 (1:400; ThermoFisher, Cat# A-11073) and goat anti-rabbit IgG Alexa Fluor 568 (1:200; ThermoFisher, Cat# A-11011) for 90 min. Sections were counterstained with DAPI (ThermoFisher, Cat# D1306), mounted on frosted slides, and coverslipped with PVA-DABCO (Sigma-Aldrich). Images were acquired using a confocal laser-scanning microscope (Olympus FV3000). Image processing was performed in ImageJ FIJI (37), including orthogonal reconstructions and maximum intensity Z-projections. Sections from the median preoptic nucleus (MnPO, Bregma +0.38) and medial preoptic area [MPA, Bregma −0.10 (33)] were imaged and anatomically matched across animals.

## 3 Results

### 3.1 Periovulatory variations in P4 and E2 in the hypothalamus, pituitary, and ovary

**Figure 2:**
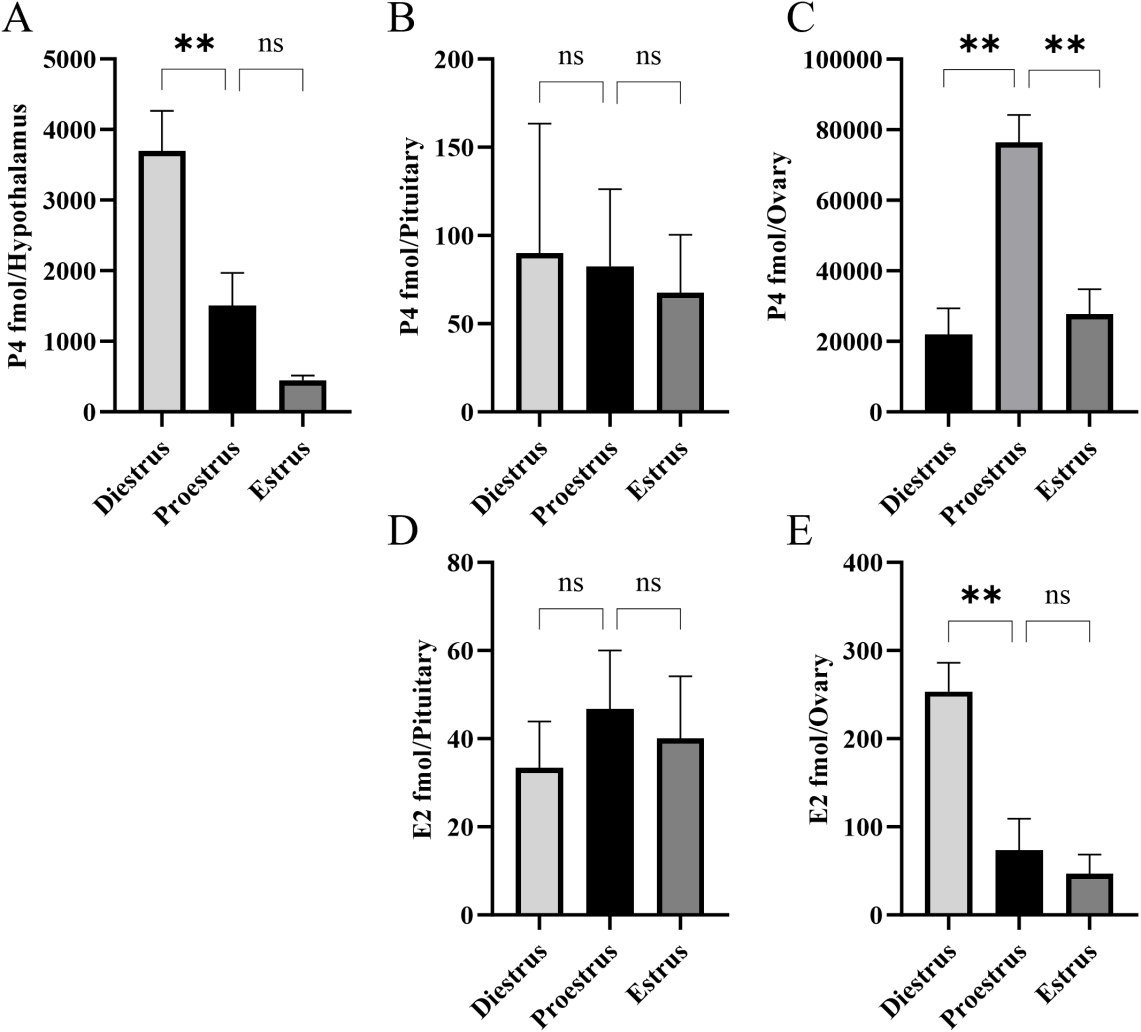
Periovulatory changes in progesterone (P4) and estradiol (E2) levels in the hypothalamus **(A)**, pituitary **(B, D)**, and ovary **(C, E)** across the estrous cycle of mice (n = 9). Mean ± SEM values are shown. Statistical significance was determined using one-way ANOVA followed by Tukey’s *post-hoc* analysis (**P < 0.01; ns: not significant). Estradiol was below the detection limit in the hypothalamus.

Progesterone (P4) was detectable in all three tissues across the estrous cycle (**Figure 2**). In the hypothalamus (**Figure 2A**), P4 levels were significantly higher at diestrus compared with proestrus and estrus. In the pituitary (**Figure 2B**), P4 concentrations remained low throughout the cycle. In the ovary (**Figure 2C**), P4 levels were approximately four-fold higher at proestrus than at diestrus or estrus. Estradiol (E2) was detected in the pituitary and ovary but remained below detection limits in the hypothalamus (**Figure 2**). Pituitary E2 (**Figure 2D**) levels were consistently low across the cycle. In the ovary (**Figure 2E**), E2 levels peaked during diestrus and declined to low values during proestrus and estrus.

### 3.2 Periovulatory variations in OXT and AVP in the hypothalamus, pituitary, and ovary

**Figure 3:**
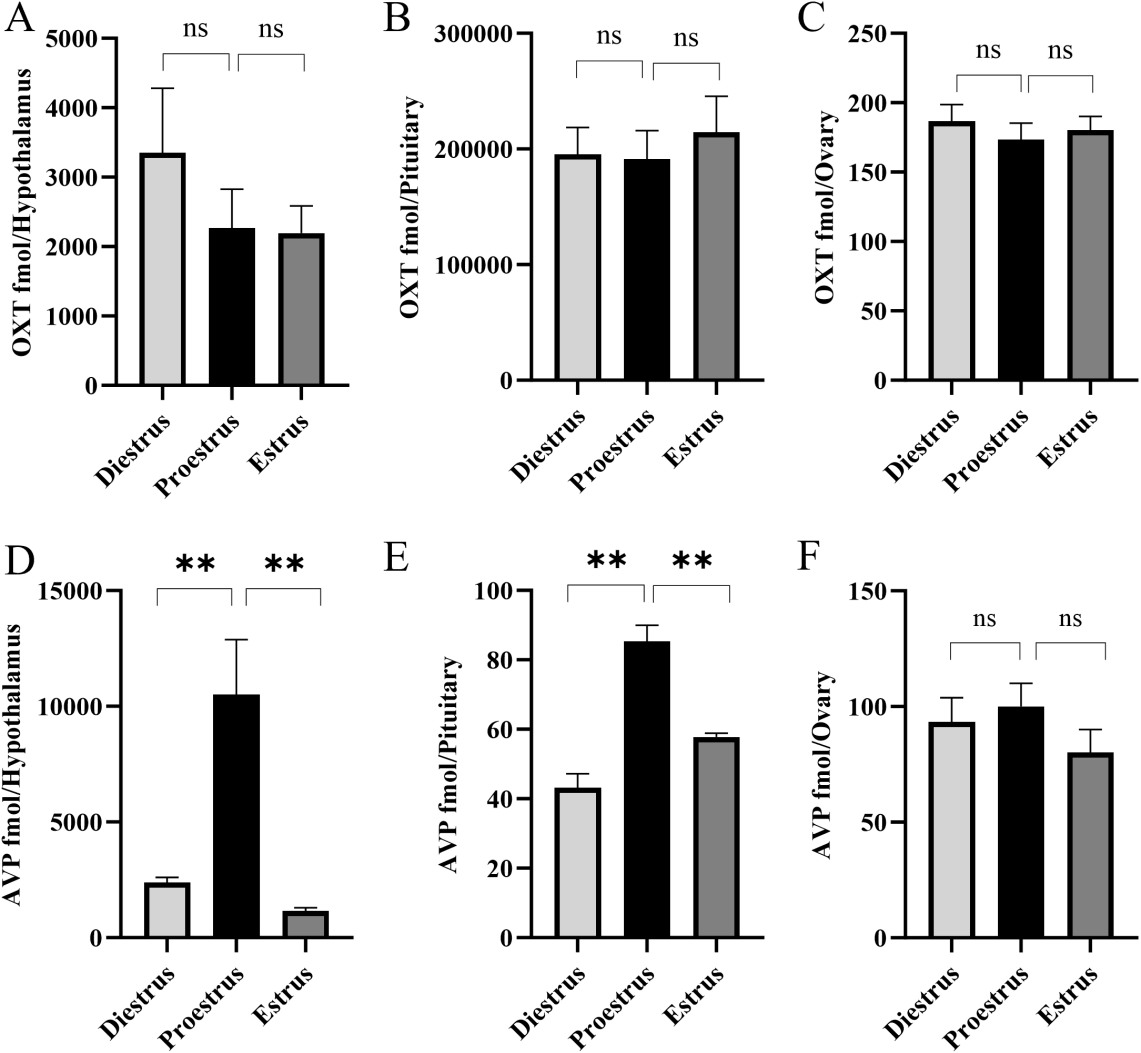
Periovulatory changes in oxytocin (OXT) and vasopressin (AVP) levels in the hypothalamus **(A, D)**, pituitary **(B, E)**, and ovary **(C, F)** across the estrous cycle of mice (n = 9). Mean ± SEM values are shown. Statistical significance was determined using one-way ANOVA followed by Tukey’s *post-hoc* analysis (**P < 0.01; ns: not significant).

Oxytocin (OXT) and arginine vasopressin (AVP) were consistently detected in all three tissues across the estrous cycle (**Figure 3**). OXT levels (**Figures 3A-C**) did not vary significantly with cycle stage. In contrast, AVP levels in the hypothalamus (**Figure 3D**) peaked at proestrus, being significantly higher than at diestrus or estrus. While AVP levels measured in the pituitary (**Figure 3E**) were lower than in hypothalamus, a similar periovulatory trend was observed. Ovarian AVP levels (**Figure 3E**) remained low and stable across stages, showing no significant differences.

### 3.3 Periovulatory variations in SN and GnRH1 in the hypothalamus, pituitary, and ovary

**Figure 4:**
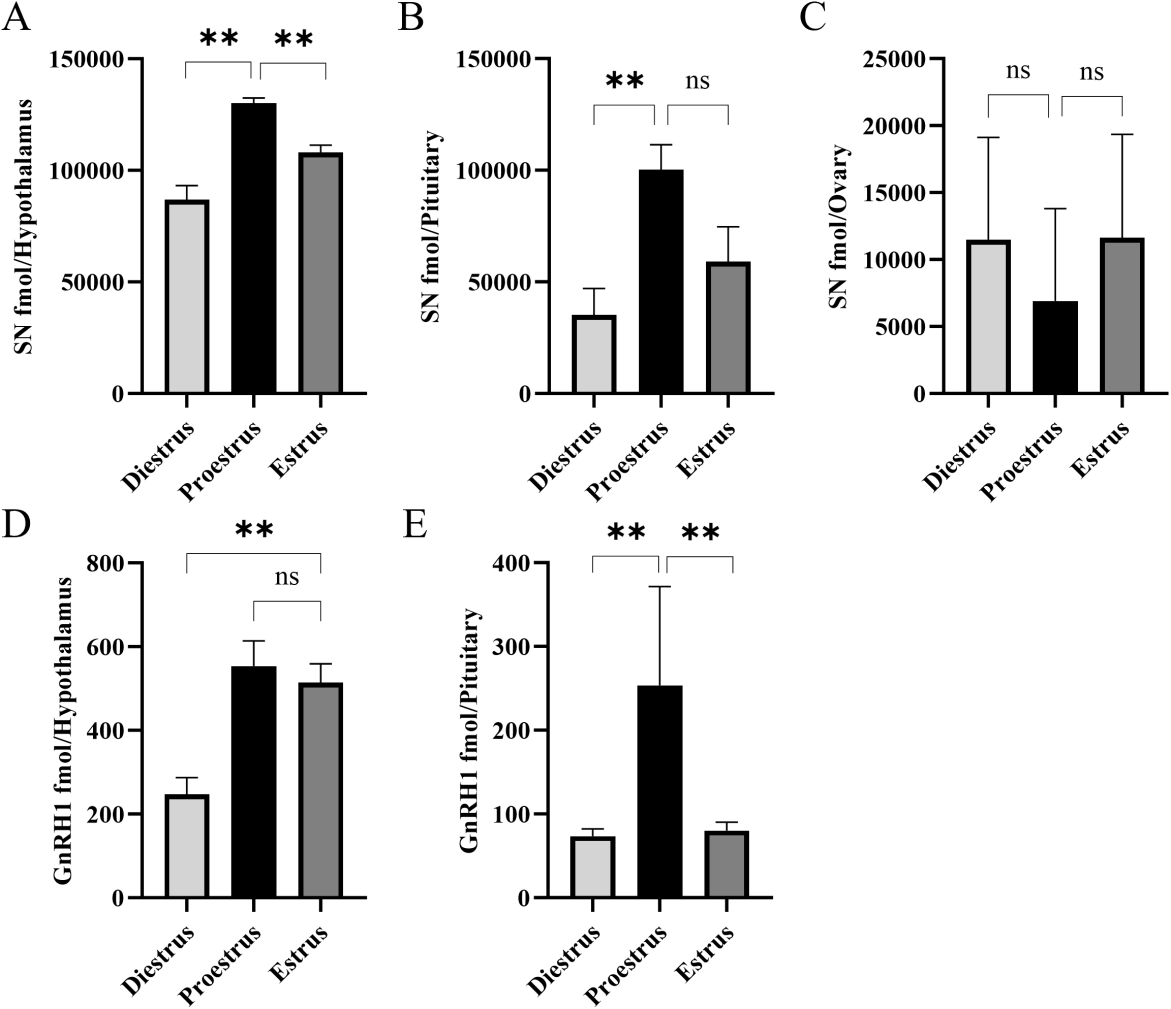
Periovulatory changes in secretoneurin (SN) and gonadotropin-releasing hormone 1 (GnRH1) levels in the hypothalamus **(A, D)**, pituitary **(B, E)**, and ovary **(C)** across the estrous cycle of mice (n = 9). Mean ± SEM values are shown. Statistical significance was determined using one-way ANOVA followed by Tukey’s *post-hoc* analysis (**P < 0.01; ns: not significant). GnRH1 was below the detection limit in the ovary. SN levels in the ovary were detectable but below the lowest point of calibration so are semi-quantitative estimations.

Secretoneurin (SN) was detected in all three tissues (**Figure 4**). In both the hypothalamus (**Figure 4A**). and pituitary (**Figure 4B**., SN levels peaked at proestrus, being significantly higher than at diestrus and estrus. In contrast, ovarian SN levels (**Figure 4C**) remained very low. GnRH1 showed a similar pattern. In the hypothalamus (**Figure 4D**), GnRH1 levels were significantly higher at proestrus and estrus compared to diestrus, while in the pituitary (**Figure 4E**) they peaked at proestrus relative to both diestrus and estrus. The GnRH1 peptide was not detected in the ovary at the times studied.

### 3.4 Regression analysis suggests positive relationship between SN and GnRH1 in the hypothalamus

Patterns in mean hormone levels suggested potential co-variation during the estrous cycle. To explore this possibility, linear regression analyses were performed comparing SN with other analytes across the three time points, with results expressed as R² values (**Table 2**). This was not applicable (N/A) to hypothalamic E2, pituitary P4, and ovarian GnRH1 because of low or non-detectable levels. Relationships were classified as absent (R² < 0.3), weak (0.3–0.5), moderate (0.5–0.7), or strong (>0.7). Among all comparisons, hypothalamic SN and GnRH1 showed the strongest association, with a moderate positive relationship (R² = 0.610; p<0.05). In the pituitary, SN displayed a weak relationship with AVP (R² = 0.403; p<0.05). In the ovary, weak relationships were observed between SN and P4 (R² = 0.321; p>0.05) and between SN and E2 (R² = 0.362; p>0.05).

**Table 2:** Linear regression coefficients of SN compared with other analytes across the estrous cycle in hypothalamus, pituitary, and ovary.

Analyte	Hypothalamus	Pituitary	Ovary
P4	0.111	N/A	0.321
E2	N/A	0.030	0.362
AVP	0.279	0.403	0.010
OXT	0.026	0.008	0.125
GnRH1	0.610	0.017	N/A

### 3.5 Secretoneurin immunoreactivity is found near preoptic GnRH1 neuronal cell bodies

**Figure 5:**
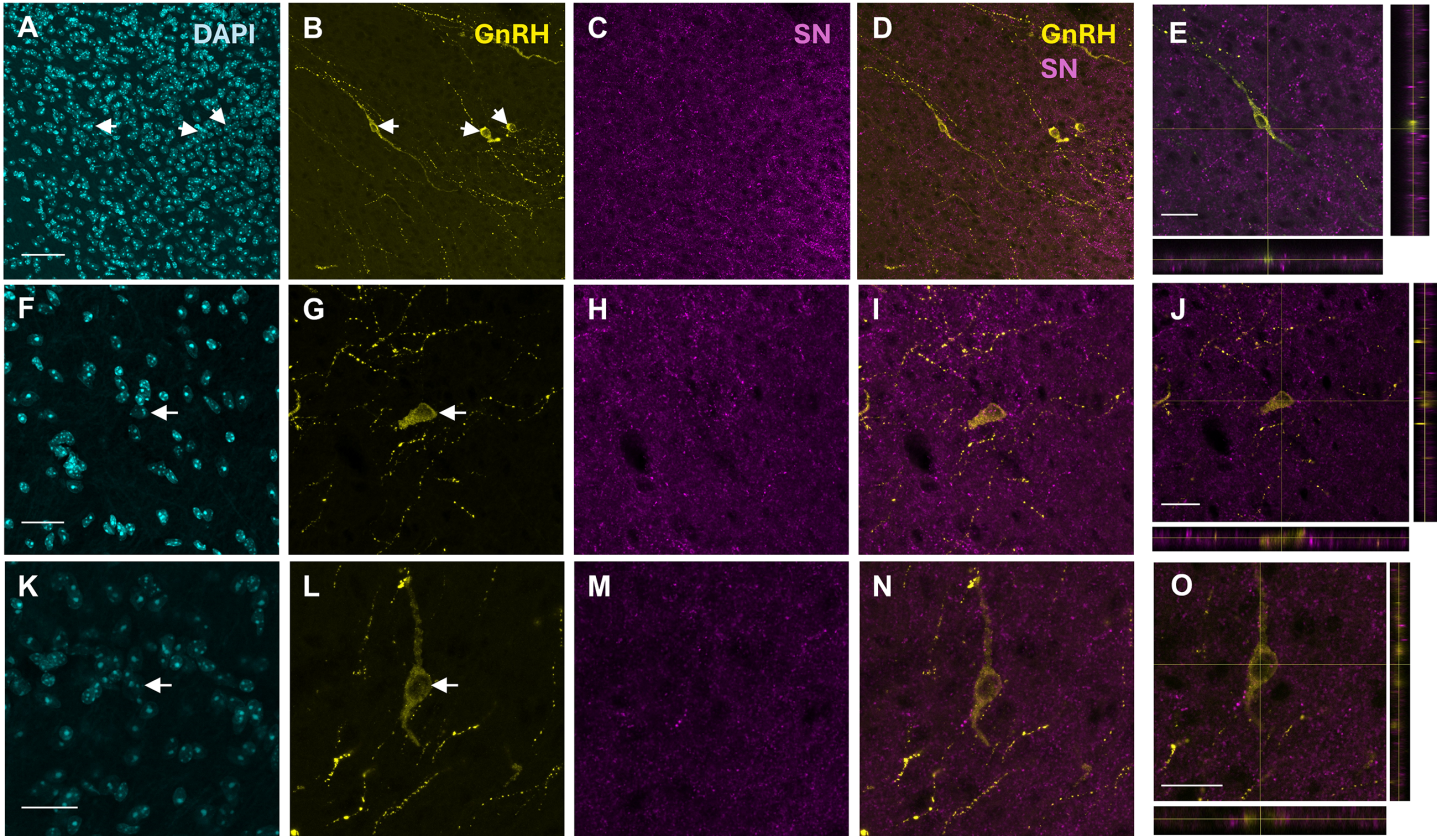
Immunocytochemical localization of GnRH1-ir and Scg2/SN-ir in female mice. Confocal microscopy images from 3 different females show DAPI nuclear staining (cyan), GnRH1 (yellow), and Scg2/SN-ir (magenta) in the median preoptic nucleus (MnPO, **A–E**) and medial preoptic area (MPA, **F–O**). Arrows indicate DAPI **(A, F, K)** positive nuclei and GnRH1-ir neurons **(B, G, L)**. Scale bars = 50 µm **(A–D)** or 25 µm **(F, I, K–N, E, J, O)**. Merged images **(D, I, N)** and orthogonal views **(E, J, O)** show the proximity of SN-ir to GnRH neurons. The GnRH neuron depicted in panel E is the one shown to the left in panel B.

To explore the neuroanatomical basis of potential SN–GnRH1 interactions in proestrus females, we examined the preoptic area (POA), given its known distribution of SN immunoreactivity in the rat hypothalamus (20) and the location of hypophysiotropic GnRH neurons (13). In all four females examined, GnRH1-immunoreactive (ir) neurons in the POA were found near SN-ir. In the median preoptic nucleus (MnPO), GnRH-ir cell bodies and fibers of passage were surrounded by SN-ir processes (**Figures 5A–E**). In the medial preoptic area (MPA), SN-ir fibers were close to GnRH1 soma–dendritic regions (**Figures 5K–O**).

### 3.6 Secretoneurin stimulates time- and dose-dependent effects on *Gnrh1* mRNA levels in GT1–7 cells

**Figure 6:**
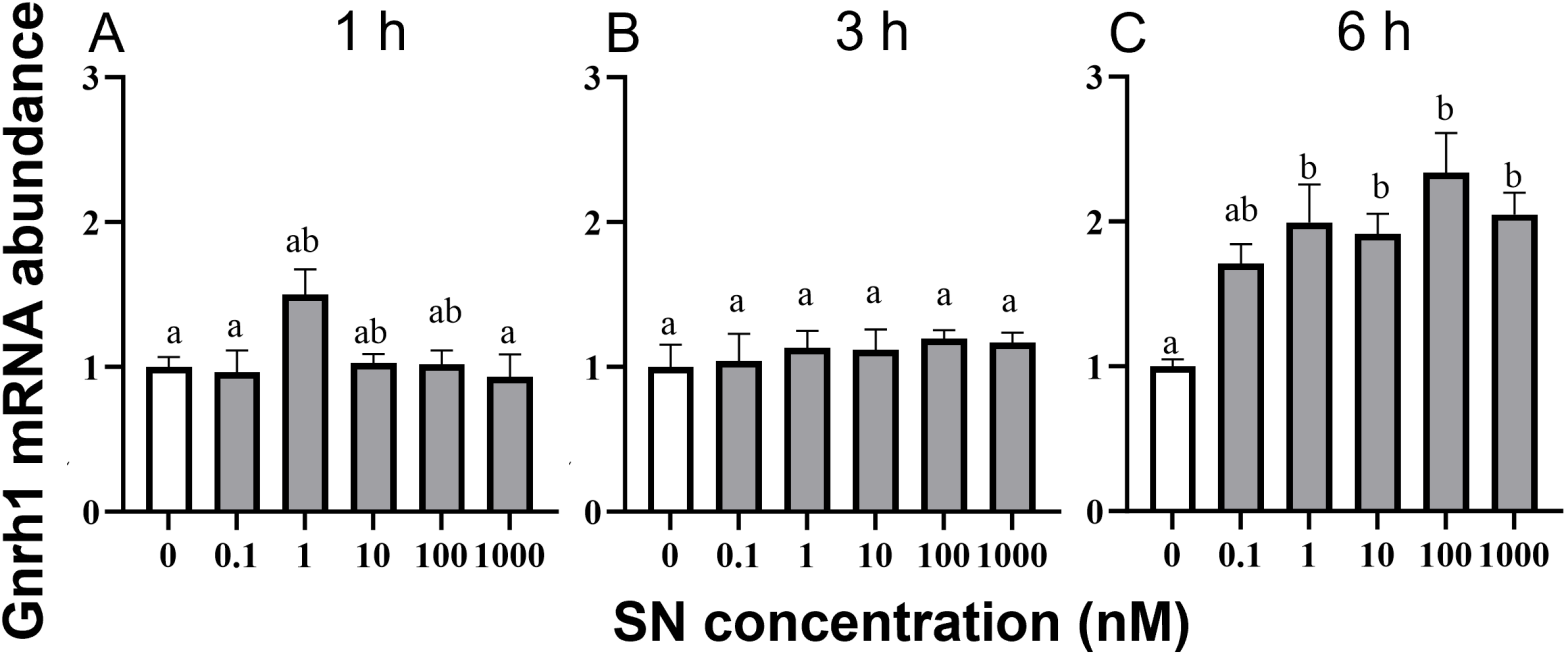
Time- and dose-response effects of SN on relative Gnrh1 mRNA abundance in mouse GT1–7 neuronal cells *in vitro*. Expression levels of *Gnrh1* were normalized to *Actb* at 1 **(A)**, 3 **(B)**, and 6 **(C)** h exposure to control (0) and graded doses of SN (0.1–1000 nM). Mean ± SEM values (n = 8) are shown. Statistical significance was determined using one-way ANOVA followed by Tukey’s *post-hoc* analysis. Different superscripts (a, b) indicate significant differences (P < 0.05).

The concomitant increase in mean hypothalamic SN and GnRH1 levels from diestrus to proestrus provided a rationale to test whether SN regulates *Gnrh1* expression in the mouse GT1–7 cell line (**Figure 6**). Incubation with SN for 1 h (**Figure 6A**) or 3 h (**Figure 6B**) had no detectable effect on *Gnrh1* mRNA levels (P > 0.05). In contrast, after 6 h SN treatment, *Gnrh1* mRNA was significantly upregulated (**Figure 6C**). A 1.7-fold increase was observed with 0.1 nM SN, although this change did not reach statistical significance (P > 0.05). Treatment with 1 nM SN induced a 2.0-fold increase (P < 0.05), while higher doses of 10, 100, and 1000 nM SN significantly increased *Gnrh1* mRNA levels by 1.9-, 2.3-, and 2.0-fold, respectively (all P < 0.05, relative to control).

### 3.7 Secretoneurin regulates intracellular signaling, secretion, and transcriptional pathways in GT1–7 cells

**Figure 7:**
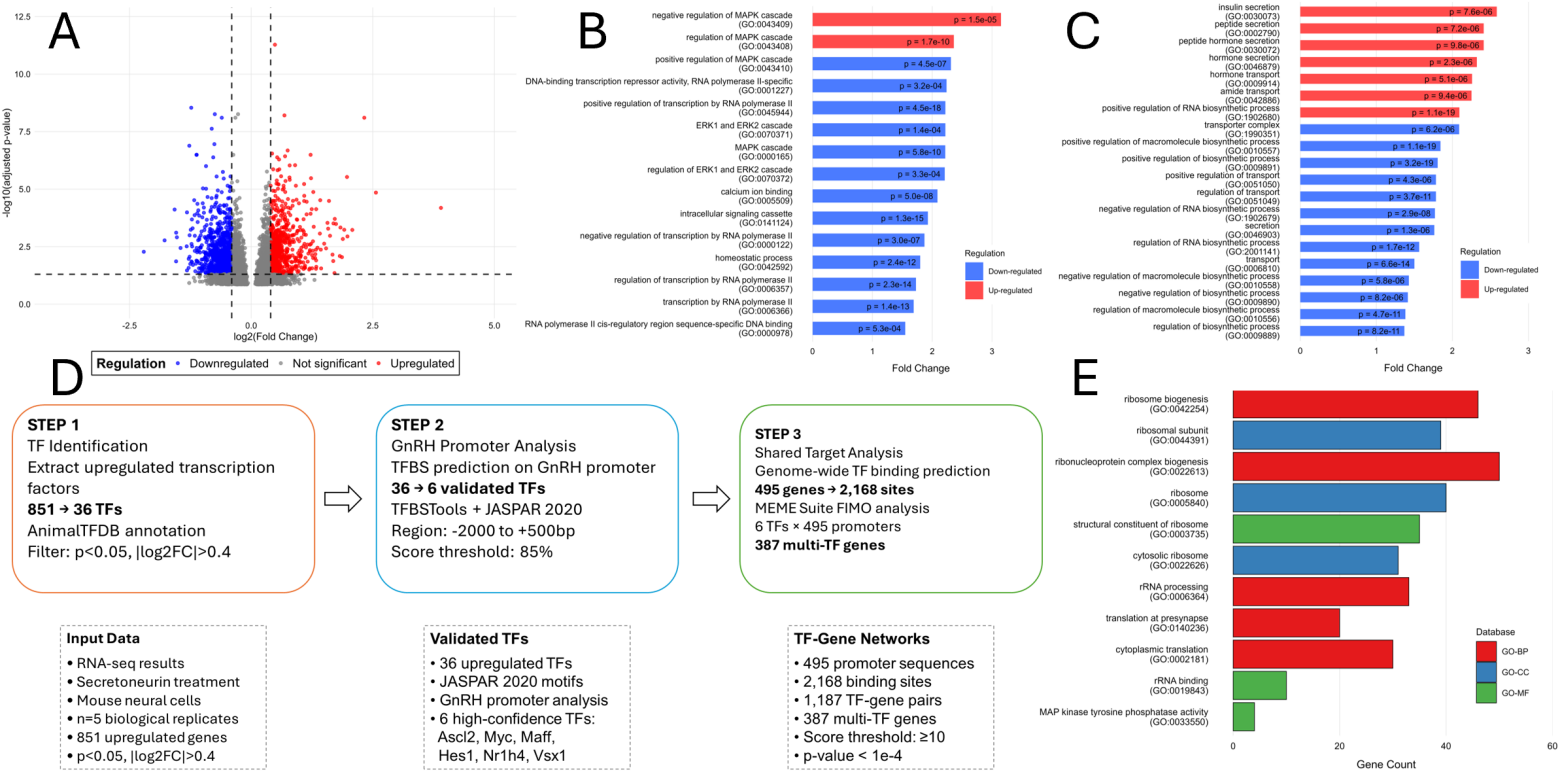
Pathway analysis of differentially regulated genes in GT1–7 cells exposed to SN. **(A)** Volcano plot displaying differential gene expression analysis results. The scatter plot shows the relationship between log2 fold change (X-axis) and statistical significance expressed as -log10(adjusted p-value) (Y-axis) for all analyzed genes. Each point represents a single gene, with red and blue points respectively representing significantly upregulated or downregulated genes, gray points show non-significant genes. Vertical dashed lines mark the fold change thresholds (|log2FC| > 0.4), while the horizontal dashed line indicates the significance threshold (adjusted p-value < 0.05). **(B)** Enrichment analysis of classic intracellular signaling pathways in differentially expressed genes. The horizontal bar chart displays the top 15 most significantly enriched signaling pathways identified through Gene Ontology (GO) analysis. Fold-change is the proportion of input DEGs that belong to each pathway term (intersection_size/query_size). Pathways are ranked by fold change (X-axis) and colored by regulation direction: red bars indicate up-regulated pathways while blue bars represent down-regulated pathways. P-values are shown for each pathway, with GO identifiers provided in parentheses for pathway identification. **(C)** Gene Ontology enrichment analysis of secretion-related biological processes. The bar plot shows the top 20 significantly enriched pathways related to cellular secretion and transport processes identified in the DEG set. Pathways are ordered by fold change values and color-coded by regulation status: red bars represent up-regulated pathways and blue bars indicate down-regulated pathways. Statistical significance is displayed as p-values for each pathway, with Gene Ontology (GO) identifiers shown in parentheses. **(D)** Transcription factor analysis pipeline for SN-induced regulation. Three-step computational workflow: (1) Identification of 36 upregulated transcription factors from 851 up regulated genes, (2) Validation of 6 high-confidence TFs through GnRH1 promoter binding analysis using JASPAR motifs, and (3) Genome-wide mapping revealing 2,168 binding sites and 387 multi-TF regulated genes. Statistical thresholds: p < 0.05, |log2FC| > 0.4 for differential expression; binding score ≥ 85th percentile and p < 1×10^−4^ for TF-DNA interactions. **(E)** Top enriched pathways across all Gene Ontology databases for multi-TF regulated genes identified from TF analysis. Gene enrichment analysis was performed on 387 genes regulated by multiple TFs using GO Biological Process (GO-BP), GO Cellular Component (GO-CC), and GO Molecular Function (GO-MF) databases. The horizontal bar chart shows the gene count for the top-ranked pathways from each database, with bars colored by database type (red: GO-BP, blue: GO-CC, green: GO-MF). Colour intensity indicates statistical significance (-log10 adjusted p-value). Pathway names are displayed with their corresponding Gene Ontology identifiers (GO IDs) for precise identification.

Given the stimulatory effect of 6 h SN treatment on *Gnrh1* mRNA in GT1–7 cells (**Figure 6C**), we next performed RNA sequencing and DEG analysis. Of the 1855 genes (**Supplementary File 1**) displaying a 1.3- fold change (log2FC>0.4), 851 were upregulated and 1004 downregulated (**Figure 7A**; red and blue points, respectively). Targeted pathway enrichment analysis of these DEGs (**Supplementary File 2**) highlighted enrichment of terms related to protein phosphorylation (**Figure 7B**). There were several GO terms related to MAPK signaling and were either up or downregulated (**Figure 7B**). Two pathways associated with ERK1 and ERK2 signaling were downregulated. Also dominating the classical signaling pathways were those related to RNA polymerase II that were downregulated. Pathways associated with calcium ion binding, intracellular signaling cascade and homeostatic processes followed this pattern of downregulation.

Both GnRH neurons and GT1–7 cells are neurosecretory, so we next examined secretion pathways. DEGs were associated with numerous secretion-related processes (**Figure 7C**). Positively regulated processes reflected SN-induced stimulation of secretory processes, for example, genes associated with insulin section, peptide secretion, hormone, and amine transport were evident. A number of downregulated pathways were associated with such processes as transporter complex, regulation of transport, and several GO terms and pathways associated with negative regulation of macromolecule biosynthesis were affected. Together, these results highlight a complex modulatory effect of SN on transcriptional and secretory regulatory programs in GT1–7 cell*s.*

We further investigated transcriptional pathway regulation (**Supplementary File 2**). Motif intersection and transcriptional regulatory network construction were used to infer key regulators and their pathways (**Figure 7D**). Motif scanning of the mouse *Gnrh1* promoter region identified 6 upregulated TFs with the potential to regulate other DEGs. Of the 36 upregulated TFs within the DEGs, ASCL2, MYC, MAFF HES1, NRLH4 and VSX1 may bind to the mouse Gnrh1 promoter. Genome-wide mapping revealed 2,168 binding sites and 387 multi-TF regulated DEGs. The analysis GO Biological Process (GO-BP), GO Cellular Component (GO-CC), and GO Molecular Function (GO-MF) databases revealed significant enrichment in ribosome-related processes, protein synthesis, and cellular component organization pathways identifying potential processes regulated by SN.

## 4 Discussion

The main motivation to carefully document changes in the classical HPG hormones was to compare them to tissue levels of the SCG2-derived peptide SN. We have now established that SN levels in the HPG axis vary in relation to two gonadal steroids and three classical neuropeptides during the mouse ovulatory cycle. These data are significant for 4 main reasons: (1) we documented periovulatory variations in P4, E2, OXT, AVP, GnRH1 simultaneously in the hypothalamus, pituitary and ovary; (2) hypothalamic SN and GnRH1 peptides increased together at proestrus; (3) SN-immunoreactive fibers are near GnRH neurons in the POA; and (4) treatment of GT1–7 neuronal cells with SN enhances *Gnrh1* mRNA, and modulates transcriptional pathways associated with biosynthetic and secretion-related processes.

### 4.1 Progesterone and estradiol

We set out to measure tissue levels of gonadal steroids and several peptides in relation to the emerging neuroendocrine regulator, SN. Tissue steroid content may not directly reflect secretion into blood, but it can reflect pools available for local actions within each tissue and provides an internally consistent metric for our multi-analyte comparisons. Progesterone and E2 are well known to regulate GnRH and LH and are usually considered in relation to blood levels and feedback actions on the hypothalamus and pituitary. Ovarian levels of P4 were highest at proestrus, being much lower at diestrus and estrus. This is somewhat different from the circulating P4 patterns reported by Wood et al. (38), who used chemiluminescent and competitive enzyme immunoassays. In the mouse, blood P4 was highest in diestrus, lowest at estrus, and intermediate at proestrus (38). On the other hand, Wall et al. (39) carefully documented serum steroid levels using MS methods, reporting peak blood P4 in proestrus that was ~10-fold higher than basal values in diestrus. This follows a similar pattern to what we observed at the tissue level: ovarian P4 was highest in proestrus, being ~3.5-fold higher than diestrus. Hypothalamic P4 progressively decreased from high diestrus levels to the lowest concentrations at estrus, thus following a similar pattern to ovarian E2 levels in this study. This is potentially important because circulating E2 can stimulate *de novo* P4 synthesis in hypothalamic astrocytes, which in turn promotes GnRH release via kisspeptin neurons (40). The role of P4 in the control of GnRH and LH is paramount (6, 41), but at the times we measured it, pituitary P4 was relatively stable.

The mammalian hypothalamus can produce estrogens because of the presence of aromatase (42), but we did not consistently detect E2 in hypothalamic samples of individual mice. While E2 was measured in the pituitary, levels were low and stable. In the ovary, E2 content was high in diestrus compared to proestrus and estrus. We report that ovarian E2 levels in diestrus are 3.5 times higher than in proestrus. This corresponds to mRNA levels of ovarian aromatase (Cyp19a1) documented for the rat across the estrous cycle, where enzyme activities were highest at proestrus (43). Moreover, Wall et al. (39) reported that serum E2 was highest in diestrus, about threefold higher than proestrus. Thus, the overall pattern of tissue E2 variation we observed is consistent with ovarian E2 regulating the GnRH pulse generator during the estrous cycle.

### 4.2 Oxytocin and vasopressin

While both OXT and AVP have been reported to have a role in the generation of the LH surge, they are rarely measured in the context of estrous cyclicity. Oxytocin levels did not vary between the studied time points. In contrast, AVP was high during proestrus in the hypothalamus and pituitary. These findings point to a potential stimulatory role for AVP in the LH surge. Some GnRH neurons express AVP receptors in female mice (16) and AVP can induce LH release (15). This was confirmed by another study that used mutant mice to reveal a critical role of AVP. The proestrus LH surge was absent when hypothalamic AVP was decreased by Clock gene mutation (44). While dissection and peptide analysis of the entire hypothalamus preclude regional specificity of the changes we report, it is noteworthy that in rodents, the AVP neurons that regulate the circadian window that permits the preovulatory LH surge localize to the suprachiasmatic nucleus. These AVP neurons regulate the LH surge through preoptic kisspeptin neurons in the female mouse (45). Magnocellular AVP populations in the supraoptic or paraventricular nuclei are unlikely to regulate the LH surge (44–46).

### 4.3 GnRH1

We measured GnRH1 peptide content in tissues, but we did not directly measure GnRH1 release. Therefore, the present data do not establish *in vivo* changes in secretion dynamics, but GnRH1 was highest in the hypothalamus and pituitary at the proestrus stage. It is well known that pulsatile GnRH1 release becomes very frequent during the proestrus stage to trigger the LH surge that subsequently leads to ovulation (1, 2). At the estrus stage, tissue GnRH1 level remained relatively high in the hypothalamus but decreased in the pituitary. This is probably because of the quick processing and release of GnRH1 around the time of the LH surge. The GnRH1 measured in the pituitary is potentially that which was released to the median eminence–portal blood system at the expected time (47). However, it must be recognized that LH cells in the pituitary can themselves produce some GnRH1, albeit relatively less than hypothalamic neurons (48).

Increased GnRH receptor mRNA and GnRH receptor binding in the pituitary prior to the LH surge coincident with increased levels of E2 in ovarian follicles drive positive feedback and the LH surge (1, 2). There is another possibility that the feedback of E2 and P4 decreased the secretion of GnRH1 at the end of the proestrus stage, which could lead to the accumulation of GnRH1 in the hypothalamus and low GnRH1 in the pituitary. GnRH1 can also be produced in the ovary to exert local paracrine actions on steroid production. While we were able to measure whole tissue levels of GnRH1, the general pattern of hypothalamic production fits Herbison’s proposed model for the regulation of the pituitary–ovarian cycle by GnRH1 in the mouse (6). The pituitary content of GnRH1 was highest when pituitary sensitivity to GnRH1 is also high (49).

### 4.4 Secretoneurin

It was found that SN was highest in both the hypothalamus and pituitary during proestrus. In other studies, SN was detected by shotgun peptidomics in mice and rat hypothalamus (50, 51). Another study in cattle reported a pubertal change in pituitary SN processing (52). In human hypothalamus, neuronal projections immunoreactive for SCG2 (https://www.proteinatlas.org/ENSG00000171951-SCG2/brain) are evident. The SN peptide was also detected at low levels in the mouse ovary, supporting the proposal that ovarian SN may regulate blood vessel formation at the time of ovulation in human, monkey, and rat ovary (53). The levels of GnRH1, AVP, and SN were all highest in the hypothalamus and pituitary at proestrus, implicating a reproductive role for SN in the mouse. In mouse LβT2 cells it is known that GnRH1 stimulates SN release, and SN in turn stimulates LH release from the cells, indicating the existence of positive autocrine control in gonadotrophs (22).

Beyond the mouse, considerable evidence indicates a fundamental role of SCG2/SN in fish reproduction (3). Genome duplication events gave rise to SCG2a and SCG2b that generate the SNa and SNb peptides, respectively (54). Phylogenetically, SNa is the more ancient form, being equivalent to tetrapod SN, while SNb is specific to teleost fish. Frameshift mutations in the *scg2* genes of zebrafish lead to decreased hypothalamic *gnrh3*, and pituitary *lhb* and *cga*, and failed spawning in ~90% of double knockout pairs (23). Moreover, SNa stimulates hypothalamic *gnrh3* coincident with increased pituitary *lhb* and *cga* and ovarian *lhcgr* and *npr* in wildtype female zebrafish (54).

Our observations prompted us to perform regression analyses to assess potential relationships between SN and other hormones in female mice. Among these, hypothalamic GnRH1 and SN were highest at the proestrus sample time and exhibited a moderate positive regression coefficient (R² = 0.61). While suggestive of a relationship, this remains correlative and requires direct *in vivo* functional studies to establish causality. For example, central SN delivery combined with measures of GnRH neuron activation, GnRH release, and the LH surge would be needed to determine whether SN is necessary and/or sufficient *in vivo*. Nonetheless, the findings in mice are consistent with prior observations in zebrafish (54). A similar analysis of dynamic hormonal changes during the zebrafish ovulatory cycle revealed that brain levels of SNa peaked concurrently with rising levels of type 3 gonadotropin-releasing hormone (Gnrh3) (R² = 0.71), at the time of the expected LH surge (54). In this case, intraperitoneal SNa injection can activate the hypothalamic–pituitary–ovarian axis and trigger ovulation within 6 h in otherwise anovulatory zebrafish females (54), but extrapolation to mammalian physiology should be made with caution until comparable studies are undertaken.

We were interested in the anatomical basis for possible SN–GnRH1 interactions in the proestrus female mouse. As in rat, the mouse median preoptic nucleus displayed SN-ir using the same antibody against the conserved mammalian SN peptide (20, 35). Double immunofluorescence revealed SN-ir near GnRH1 cell bodies in the median and medial preoptic area in all four female brains examined. Although samples were limited to proestrus females, and synaptic connectivity remains to be demonstrated, these findings raise the possibility of direct anatomical interactions. While the origin of these SN fibers is unknown, such inputs to GnRH neurons are consistent with the multiplicity of regulatory inputs to them (1, 2, 14, 55). These results lay the foundation for more extensive neuroanatomical assessments of SN in the future.

Transcriptomic responses were assessed in a well-characterized GnRH neuronal model cell line rather than in hypothalamic tissue. GT1–7 cells provide a controlled system to identify SN-responsive programs in GnRH-like neurons. Time- and dose-response analysis indicates that 1–1000 nM SN increase *Gnrh1* mRNA by ~2-fold by 6 h of incubation. This is consistent with increased *Gnrh1* mRNA by other known stimulators of GT1–7 cells, such as kisspeptin (56) and nesfatin-1 (27). Such increases in *Gnrh1* provides evidence for a transcriptional response, but it cannot be assumed to translate to increased GnRH peptide secretion. We then performed RNASeq on controls and those GT1–7 cells with the highest level of *Gnrh1* mRNA (100 nM SN exposure group). Targeted analysis and GO term associations provide a mechanistic basis for transcriptional effects of SN. We report that several MAPK and ERK1/2-related pathways presented as both up- and downregulated in the DEG set. Calcium ion binding, homeostatic processes and intracellular signaling presented as downregulated in the pathway analysis. Previous data indicate that 10–180 min exposure to SN activates the protein kinase A (PKA) and cAMP-induced ERK signaling pathways in the LH-secreting mouse LβT2 pituitary cell line (22). The effects of prolonged exposure to SN were not investigated in that study. Taken together, data from the GT1–7 and LβT2 cell lines implicate the MAPK signaling pathway in SN action, though with temporal complexity.

Secretoneurin also enhances proliferation of mouse endothelial cells through activation of the PI3K/Akt and MAPK signaling pathways (57), and the SNb isoform is required for central artery formation in 2-day-old zebrafish via similar mechanisms (58). Despite substantial investigation, the specific SN receptor has not yet been identified. Findings from SN radioligand binding studies, along with activation of signaling pathways responsive to cholera and pertussis toxins, elevations in cyclic AMP (cAMP), and downstream engagement of PKA and ERK, suggest the involvement of a G-protein-coupled receptor (59). Alternative models propose that SN may undergo receptor-mediated internalization, enabling intracellular interactions with Ca^2+^/calmodulin (CaM) and CaM-dependent protein kinase IIδ, which may modulate certain cellular activities while enhancing phosphorylation of ryanodine receptors in cardiomyocytes (60). The N-terminal, non-SN portion of SCG2 functionally interacts with leukocyte immunoglobulin-like receptor B4 (LILRB4) on monocytic cells to drive intracellular tyrosine phosphorylation (61). This interaction specifically involves amino acids 31–179 of full-length human SCG2, which immediately precede the first dibasic cleavage site generating SN (SCG2 182-214; P13521.2). In the context of reproductive neuroendocrinology, these mechanisms remain speculative, and the full spectrum of SCG2/SN-activated signaling has yet to be fully elucidated.

This led us to determine candidate regulatory relationships following 4 criteria: (1) transcription factor upregulation in the DEG list; (2) predictions of high-confidence binding to the mouse *Gnrh1* promoter; (3) statistically significant target gene predictions; and (4) biological pathway consistency. The analysis suggests that ASCL2 MYC, MAFF HES1, NRLH4 and VSX1 may bind to the mouse *Gnrh1* promoter. Other known TFs that regulate the mouse *Gnrh1* gene include class-C SOX transcription factors, VAX1, and cFOS (62, 63), but were not modulated by SN in GT1–7 cells. There were 387 multi-TF regulated DEGs linked to pathways significantly enriched in ribosome-related processes, protein synthesis, and cellular component organization pathways. Together with our targeted pathway analyses, this implies that SN has an impact on phosphorylation and secretion and supports protein manufacture in GT1–7 GnRH neuronal cells. Because these analyses are based on transcriptomic enrichment and motif predictions, they should be interpreted as candidate mechanisms that require targeted validation at the protein and signaling level.

## 5 Conclusion and perspective

Periovulatory neuroendocrine regulation in mice involves coordinated changes in multiple hormonal systems. Ovarian P4 and E2 provided the expected steroidal cues across the cycle, while hypothalamic AVP peaked during proestrus in a manner consistent with its proposed role in LH surge timing. Within this framework, GnRH1 showed cycle-dependent changes in both the hypothalamus and pituitary. SN displayed a concurrent rise with GnRH1 and AVP in hypothalamus and pituitary, and SN-ir was observed near GnRH1 neurons in the preoptic area. *In vitro*, SN stimulated *Gnrh1* mRNA in GT1–7 cells in a time- and dose-dependent manner. Transcriptomic profiling of these SN-treated cells identified DEGs enriched for transcriptional and secretory processes. We performed TF identification and regulatory network analysis, which supports the proposal that SN regulates key cellular processes in GnRH1 neurons. While these findings suggest possible mechanisms of action, their functional significance for fertility remains to be tested. These results support the proposal that SN is a component of the periovulatory neuroendocrine network, and they provide a strong foundation for future *in vivo* studies on GnRH neuronal activation and peptide secretion to define its precise role in mice and conservation across vertebrates.

## Data Availability

The original contributions presented in the study are included in the article/**Supplementary Material**. Further inquiries can be directed to the corresponding author/s.
