# Supplementary material for: Periovulatory neurohormone dynamics reveal an association between secretoneurin and GnRH across the mouse estrous cycle
Source: Front Endocrinol (Lausanne). 2026 Jan 23;16:1708570. doi: 10.3389/fendo.2025.1708570 (PMC12875923; doi:10.3389/fendo.2025.1708570)
Supplement: Supplementary file 2 [file DataSheet2.docx]

**Supplementary File 2**

**Periovulatory neurohormone dynamics reveal an association**

**between secretoneurin and GnRH across the mouse estrous cycle**

Chunyu Lu^1*^, Di Peng,^1*^ Kevin Smith^2^, Chinelo Uju^3^, Suraj Unniappan^3^, Paula Duarte-Guterman^4^, Nafissa Ismail^2^, Vance L. Trudeau^1+^ (Final order to be discussed)

^1^Department of Biology and ^2^School of Psychology, University of Ottawa, Ottawa, ON K1N 6N5, Canada

^3^Department of Veterinary Biomedical Sciences, Western College of Veterinary Medicine, University of Saskatchewan, Saskatoon, SK S7N 5B4, Canada

^4^Department of Psychology, Brock University, St. Catharines, ON L2S 3A1, Canada

*Contributed equally

^+^Correspondence: trudeauv@uottawa.ca

**Detailed Methods for Transcription Factor Identification and Regulatory Network Analysis**

**1. Transcription Factor Identification and Screening**

Data Source: Based on RNA-seq data from mouse neural cell lines treated with secretoneurin (n=6, 100nM, 6 hours), with differential expression analysis completed.

Transcription Factor Annotation: - Database: Mouse transcription factor database (AnimalTFDB v3.0) - Filtering criteria: significance == “Upregulated”, p-value < 0.05, |log2FC| > 0.4 - Identified upregulated transcription factors for subsequent analysis [1].

Quality Control: Examined the expression status of five known GnRH regulatory factors (CREB1, EGR1, SP1, FOS, JUN) in the original dataset. None of these classical regulatory factors were detected in the differentially expressed genes, suggesting that secretoneurin regulates GnRH through novel transcriptional regulatory mechanisms.

2. GnRH Promoter Analysis and Transcription Factor Validation

Sequence Acquisition: - Target gene: Gnrh1 (ENSMUSG00000015812) - Promoter region: 2000 bp upstream to 200 bp downstream of transcription start site (-2000 to +200 bp) - Reference genome: GRCm39 (mm39)

Transcription Factor Binding Site Prediction: - Tool: TFBSTools package [2] - Database: JASPAR 2020 vertebrates collection [3] - Parameter settings: - Minimum score threshold: 85% of maximum possible score - Strand scanning: Both positive and negative strands analyzed - PWM matrix normalization: Converted to log-odds matrices

Validated Transcription Factor Selection Criteria: 1. Successfully matched with JASPAR motif database 2. Binding sites detected in GnRH promoter region 3. Binding score ≥ 85% threshold 4. Transcription factor itself significantly upregulated in experiment

Final Validation Set: 6 validated transcription factors identified: - Ascl2 (bHLH family): Neural development regulator - Myc (bHLH family): Cell proliferation and metabolism control - Maff (bZIP family): Transcriptional activation and stress response - Hes1 (bHLH family): Notch signaling downstream effector - Nr1h4 (Nuclear receptor family): Metabolic regulation - Vsx1 (Homeobox family): Neuronal differentiation control

**3. Genome-wide Shared Target Gene Analysis**

Technical Approach: Due to technical limitations of TFBSTools in large-scale analysis, MEME Suite was employed for transcription factor binding site prediction.

Sequence Preparation: - Analysis scope: 495 upregulated genes (excluding GnRH) - Promoter definition: -2000 to +500 bp from transcription start site - Sequence extraction: Using BSgenome.Mmusculus.UCSC.mm39

FIMO Analysis Parameters: - Tool: MEME Suite FIMO v5.4.1 - Runtime environment: Docker container (memesuite/memesuite) - Statistical threshold: p-value < 1e-4 - Scanning mode: Both strand scanning - Motif database: 6 validated transcription factor motifs extracted from JASPAR 2020 [4].

Binding Site Classification System: - Ultra-reliable: Score ≥15, p-value < 1e-6 (direct regulatory relationships) - High-confidence: Score 12-15, p-value < 1e-5 (strong regulatory evidence) - Supported: Score 10-12, p-value < 1e-4 (likely regulatory connections) - Weak: Score < 10 (low confidence)

**4. Transcription Factor-Target Gene Network Construction**

Regulatory Complexity Quantification: - Single regulation: Genes bound by 1 transcription factor - Cooperative regulation: Genes co-bound by 2-3 transcription factors - Complex regulation: Regulatory hub genes bound by 4+ transcription factors

Network Quality Validation: 1. Coverage: 94.7% of analyzed genes successfully detected transcription factor binding 2. Specificity: All 6 validated transcription factors identified target genes 3. Biological relevance: Transcription factor family distribution matches expected patterns

**5. High-Confidence Gene Filtering**

Filtering Strategy: Selected multi-transcription factor regulated genes as high-confidence target gene set, based on the following logic: - Single transcription factor binding may contain false positives - Multiple transcription factor cooperative binding suggests important regulatory nodes - Combinatorial regulation more likely represents true biological regulatory relationships

Filtering Criteria: - Transcription factor count: ≥2 validated transcription factors binding - Binding quality: At least 1 high-confidence or ultra-reliable binding site included - Expression consistency: Gene itself significantly upregulated in experiment

Final Gene Set: 387 multi-transcription factor regulated genes, with an average of 2.8 transcription factors regulating each gene.

**6. Pathway Enrichment Analysis**

Gene ID Conversion: - Input: 387 Ensembl gene IDs (ENSMUSG format) - Conversion tool: bitr function from org.Mm.eg.db package - Output: Entrez Gene IDs for enrichment analysis - Conversion efficiency: >95% success rate

Enrichment Analysis Parameters: - Tool: clusterProfiler v4.0+ [5] - Statistical method: Hypergeometric test + Benjamini-Hochberg correction - Significance threshold: p.adjust < 0.05, q-value < 0.2 - Gene set size: Minimum 10 genes, maximum 500 genes

Analysis Databases: 1. GO Biological Process: Biological process functional annotation 2. GO Molecular Function: Molecular function annotation 3. GO Cellular Component: Cellular component localization 4. KEGG Pathways: Metabolic and signaling pathways 5. Reactome Pathways: Reactome pathway database

**7. Result Validation and Quality Control**

Computational Validation: 1. Known regulator recovery: Validation of literature-reported GnRH regulatory factors in prediction results 2. Motif conservation analysis: Sequence conservation validation of high-score binding sites 3. Expression correlation: Correlation analysis between transcription factor and target gene expression levels

Biological Validation Metrics: 1. Transcription factor family distribution: Reasonable distribution of bHLH, bZIP, nuclear receptor families 2. Pathway consistency: Biological relevance of enriched pathways to secretoneurin-GnRH regulation 3. Literature support: Literature evidence support for key prediction results

Statistical Robustness: - Permutation test: Background distribution of enrichment for random gene sets - Sensitivity analysis: Impact of different threshold parameters on results - Reproducibility assessment: Stability of core findings under different parameter settings

**8. Key References for Transcription Factor Identification and Regulatory Network Analysis**

1. Hu H, Miao YR, Jia LH, Yu QY, Zhang Q, and Guo AY (2019). AnimalTFDB 3.0: a comprehensive resource for annotation and prediction of animal transcription factors. Nucleic Acids Res. 47(D1):D33–D38. doi: 10.1093/nar/gky822
2. Tan G, and Lenhard B (2016). TFBSTools: an R/bioconductor package for transcription factor binding site analysis. Bioinformatics 32(10):1555–1556. doi: 10.1093/bioinformatics/btw024
3. Fornes O, Castro-Mondragon JA, Khan A, van der Lee R, Zhang X, Richmond PA, et al. (2020). JASPAR 2020: update of the open-access database of transcription factor binding profiles. Nucleic Acids Res. 48(D1):D87–D92. doi: 10.1093/nar/gkz1001
4. Grant CE, Bailey TL, and Noble WS (2011). FIMO: scanning for occurrences of a given motif. Bioinformatics 27(7):1017–1018. doi: 10.1093/bioinformatics/btr064
5. Wu T, Hu E, Xu S, Chen M, Guo P, Dai Z, et al. (2021). clusterProfiler 4.0: a universal enrichment tool for interpreting omics data. Innovation (Camb). 2(3):100141. doi: 10.1016/j.xinn.2021.100141
